# The Nutritional Profile of Spanish Beverages: A Comparative Evaluation of the Original and Updated Nutri-Score Algorithm

**DOI:** 10.3390/nu17091521

**Published:** 2025-04-30

**Authors:** Sara de las Heras-Delgado, Sangeetha Shyam, Lucía Iglesias-Vázquez, Nadine Khoury, Jordi Salas-Salvadó, Nancy Babio

**Affiliations:** 1 Universitat Rovira i Virgili, Departamentde Bioquímica i Biotecnologia, Unitat de Nutrició Humana, Grup ANUT-DSM, 43201 Reus, Spain; sara.delasheras@urv.cat (S.d.l.H.-D.); sangeetha.shyam@urv.cat (S.S.); lucia.iglesias@urv.cat (L.I.-V.); nadine.khoury@urv.cat (N.K.); 2 Institut d’Investigació Sanitària Pere Virgili (IISPV), 43204 Reus, Spain; 3 Centro de Investigación Biomédica en Red Fisiopatología de la Obesidad y la Nutrición (CIBEROBN), Institute of Health Carlos III, 28029 Madrid, Spain

**Keywords:** Nutri-Score, nutrient profiling model, beverage, public health, nutritional labeling

## Abstract

**Background**: In response to criticism and limitations of the Nutri-Score Nutrient Profiling Model (NS-NPM), the algorithm was updated in 2023. However, its impact on beverage classification remains partially assessed. **Objective**: This study aimed to compare the nutritional profiles of beverages marketed in Spain using the original and updated NS-NPM algorithms. **Methods**: Nutritional data for 3432 beverages in the “Drink Base” database were analyzed using both the 2015 (original) and 2023 (updated) NS-NPM versions. **Results**: The 2023 update showed significant changes compared to the 2015 version. Updated scores particularly increased for artificially sweetened beverages (+190.3%), milkshakes (+98.9%), nut-based beverages (+343.9%), cereal-based beverages (+651.3%), and the mix of plant-based beverages (+733%), leading to a less healthy classification. Conversely, scores decreased for fruit juices (−12.7%) and alcohol-substitute beverages (−8.2%), while legume-based beverages maintained their classification with minimal score variation (−1.4%), raising questions about the treatment of free sugars. The remaining beverage categories experienced score changes that did not alter their classification. **Conclusions**: The 2023 NS-NPM algorithm improves beverage classification by refining the differentiation of sugar-sweetened and artificially sweetened beverages, improving consumer guidance. While it increases discrimination, challenges remain in the classification of plant-based beverages and fruit juices. These findings highlight Nutri-Score’s impact on industry reformulation and its potential as a public health tool to promote healthier beverage choices. This study provides novel evidence on how the updated Nutri-Score algorithm may influence consumer perception and food policy in the Spanish context.

## 1. Introduction

In recent years, diets have shifted toward increased consumption of unhealthy, highly processed foods and beverages [1]. In Spain, the non-alcoholic beverage market includes a wide variety of products, such as soft drinks, fruit juices, plant-based alternatives, and alcohol substitutes. This diversification of the beverage market has important implications for population-level sugar intake and health outcomes. In 2022, Spanish households spent approximately EUR 2000 million on purchasing non-alcoholic beverages [2], reflecting a high demand for these products. Non-alcoholic beverages contribute up to 16% of total energy intake and over one-third of total sugar intake [3]. They have been linked to increased risks of weight gain, obesity [4], diabetes [4,5], and cardiovascular diseases [5,6]. Recent global reports continue to warn about the contribution of sugary beverages to obesity and non-communicable diseases, reinforcing the need for effective labeling systems [7,8]. Given that sugar-sweetened beverages are a primary contributor to free sugar intake [9], their classification under Nutri-Score has direct implications for public health strategies aimed at reducing excessive consumption.

In response to these health concerns, public health strategies encourage consumers to make healthier dietary choices [9]. One such strategy is the use of nutrient profiling models (NPMs), scientific tools designed to classify foods based on their nutritional composition to support consumer decision-making and public health policies. Among these, front-of-pack (FOP) labeling systems, such as warning labels [10] and the Nutri-Score [11], have been implemented to guide consumer choices at the point of purchase. Nutri-Score, developed in 2015, is a widely adopted FOP labeling system that classifies products into five categories based on an algorithm that evaluates the nutritional composition, balancing favorable components (e.g., protein and fiber) and unfavorable ones (e.g., sugar and saturated fat) [12]. However, concerns about the accuracy of Nutri-Score’s classification, particularly regarding certain food groups, led the Nutri-Score Scientific Committee to refine the algorithm for both food and beverages [13].

The 2023 Nutri-Score Nutrient Profiling Model (NS-NPM) was published in 2022 for food and in 2023 for beverages [14,15]. The updated version incorporates changes to align Nutri-Score more closely with food-based dietary guidelines, including considerations for non-nutritive sweeteners and the classification of plant-based beverages and milk as a beverage. Despite updates to the Nutri-Score algorithm, little is known about its impact on beverage classification, particularly in Spain. Based on previous findings in other European countries [16,17,18], we hypothesized that the updated Nutri-Score would downgrade milk, milkshakes, sweetened plant-based beverages, and artificially sweetened products while having minimal effect on fruit juices and provide better discrimination within categories.

This study aims to address that gap by comparing the classification of beverages marketed in Spain using the original (2015) and updated (2023) Nutri-Score algorithms. It provides new evidence on how the updated model affects a key food category with high public health relevance. By focusing on the Spanish market, our findings complement previous evaluations conducted in other European countries and offer insights into the potential impact of algorithm updates on consumer information and industry reformulation.

## 2. Materials and Methods

### 2.1. Study Design

We conducted a descriptive and comparative study to assess how the updated Nutri-Score algorithm classifies Spanish beverages compared to the original version.

### 2.2. Study Products

A total of 3432 non-alcoholic beverages were included in the analysis. These products were selected from two sources. First, we utilized the “Drink Base” database [19], which compiles nutritional information from a total of 9049 beverages available in the Spanish market (3237 non-alcoholic, 142 mineral water, and 5670 alcoholic beverages). The “Drink Base” database, updated every five years, includes all beverages marketed in Spain for which nutritional information could be obtained and verified. This information was sourced from product technical sheets or data provided by manufacturers and/or distributors through their official websites, online retailers, or nutrition labeling.

**Figure 1:**
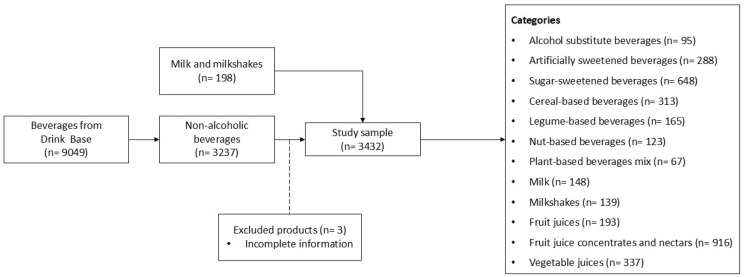
Flowchart of the selection and inclusion process of beverages from the “Drink Base” database and supermarket sample.

Second, we included a random sample of milk and milkshakes from five major Spanish supermarket chains (Alcampo [20], Carrefour [21], Consum [22], Mercadona [23], and Supermercado Dia [24]), which were not part of the “Drink Base” database. To ensure a representative sample, beverages were selected from relevant product categories in online or physical stores of each supermarket, using a systematic random sampling method based on product listing order. The selected categories represent the most consumed non-alcoholic beverages in Spain, according to national food consumption data [25]. Additionally, the included supermarket chains are among the largest and most widely distributed in the country, accounting for most of the grocery retail market share. Therefore, the selected products are considered representative of the beverage options most frequently available to Spanish consumers. The selection process is illustrated in Figure 1.

Beverages were classified into 12 categories based on previous research on FOP labeling systems and nutrient profiling models. The categories included alcohol-substitute beverages, artificial sweetened beverages, sugar-sweetened beverages, cereal-based beverages, legume-based beverages, nut-based beverages, plant-based beverages mix, milk, milkshakes, fruit juices, concentrates and nectars, and vegetable juices. The classification criteria included ingredient composition, the presence of added sugars, and processing methods. Beverages containing both added sugar and artificial sweeteners were classified according to their predominant characteristic. If a product contained both, it was categorized as an “artificially sweetened beverage” if non-nutritive sweeteners were present at a significant level, as indicated in the ingredients list.

Other flavored or fermented dairy beverages were not included in this study as these products were not prominently represented in the main Spanish supermarket chains screened.

### 2.3. NS-NPM Index Computation

The Nutri-Score for each product was calculated using the official Excel spreadsheet provided by Santé Publique France [26].

Nutritional values were extracted from mandatory nutrition labeling on product packages in accordance with Regulation (EU) No. 1169/2011 [27]. The extracted data included energy (kcal/kJ), carbohydrates (g), sugars (g), total fat (g), saturated fats (g), salt (g), and protein (g). Following a conservative approach, fiber values were imputed as zero when unavailable as fiber declaration is not mandatory under European regulations [28]. This conservative approach assumes the lowest possible fiber content, minimizing the risk of overestimation. While an alternative approach could involve inputting the category mean, zero assumption ensures a more cautious evaluation of products.

The list of ingredients was used to determine the presence or absence of specific components, including fruits, vegetables, legumes, the oil of nuts, canola, walnut and olive, and non-nutritive sweeteners [26].

#### 2.3.1. Original Version (2015 NS-NPM)

The original Nutri-Score algorithm classified foods and beverages separately [12]. In this version, plant-based beverages, milk, and milkshakes were categorized regular food rather than beverages. The score was calculated based on the nutrient content and ingredient list per 100 mL of product. Each identified nutrient component was assigned a score based on whether it was considered favorable (e.g., protein, fiber, or key food ingredients) or unfavorable (e.g., energy, sugar, salt/sodium, and saturated fat) (see Supplemental Figure S1). Favorable elements included protein (0 to 5 points; ≤1.6 to >8 g), fiber (0 to 5 points; ≤0.7 to >3.5 g), and the percentage of fruits, vegetables, legumes, the oil of nuts, and canola, walnut, and olive, which were assigned 0 points for ≤40%, 2 points for >40%, 4 points for >60%, and 10 points for >80%.

Unfavorable elements included energy (0 to 10 points; ≤0 to >270 kJ), sugar (0 to 10 points; ≤0 to >13.5 g), saturated fat (0 to 10 points; ≤1 to >10 g), and sodium (0 to 10 points; ≤90 to >900 mg).

Specifically for plant-based beverages, milk and milkshakes, the percentage of fruits, vegetables, legumes, and the oil of nuts, canola, walnut, and olives were assigned as follows: 0 points for ≤40%, 1 point for >40%, 2 points for >60%, and 5 points for >80%. Regarding energy, the scoring ranged from 0 to 10 points, with thresholds from ≤335 to >3350 kJ or ≤80 to >800 Kcal. Similarly, sugar content was scored from 0 to 10 points, with thresholds from ≤4.5 to >45 g (see Supplemental Figure S2).

The final score was calculated by subtracting the sum of unfavorable elements from the sum of favorable elements, resulting in a theoretical range from −20 (healthiest) to 40 (least healthy).

Beverages were classified into five categories: A (dark green) for water, B (light green) for scores < 1 to 1, C (yellow) for scores 2 to 5, D (orange) for scores 6 to 9, and E (red) for scores 10 and above (see Supplemental Figures S1 and S2).

#### 2.3.2. Updated Version (2023 NS-NPM)

The 2023 NS-NPM [16,29] introduces two key changes: (1) classifying plant-based beverages, milk and milkshakes as beverages and (2) adding non-nutritive sweeteners as an unfavorable component, assigning 0 points if absent and 4 points if present.

Additionally, the updated scoring system includes revised cut-off points. Favorable elements included protein (0 to 7 points; ≤1.2 to >3 g), fiber (0 to 5 points; ≤3 to >7.4 g), and the percentage of fruits, vegetables, legumes, the oil of nuts, canola, walnut and olive, which were assigned 0 points for ≤40%, 2 points for >40%, 4 points for >60%, and 6 points for >80%. Unfavorable elements included energy (0 to 10 points; ≤30 to >390 kJ), sugar (0 to 10 points; ≤0.5 to >11 g), and salt (0 to 20 points; ≤0.2 to >4 g). The final score now ranges from −18 to 54 points, with corresponding redefined category thresholds (see Supplemental Figure S3).

The thresholds for Nutri-Score FOP labeling categories have also been adjusted. While Category A remains unchanged, the thresholds for the other categories have been modified as follows: Category B (light green, <2 to 2), Category C (yellow, 3 to 6), Category D (orange, 7 to 9), and Category E (red, ≥10).

### 2.4. Statistical Analyses

Due to the non-normal distribution of variables across most beverage categories, quantitative data are reported as medians and interquartile ranges, while qualitative variables were expressed as percentages (n). The non-parametric Wilcoxon signed-rank test was applied to determine statistically significant differences in NS-NPM scores between the original and updated Nutri-Score algorithms. This test is appropriate for paired ordinal or continuous data that are not normally distributed, allowing a comparison of within-product differences across versions.

Spearman correlation was used to assess the consistency of ranking between the 2015 and 2023 Nutri-Score versions as it measures monotonic relationships without assuming normality or linearity. This analysis was useful to evaluate whether the relative positioning of beverages remained stable across versions.

Additionally, relative variation (%) in scores was calculated using the formula: ((2023 NS-NPM score − 2015 NS-NPM score)/2015 NS-NPM score) × 100.

Box plots were used to visualize the distribution of NS-NPM scores across beverage categories for both algorithm versions, depicting the median, 25th and 75th percentiles, and Nutri-Score categories.

To compare the distribution of Nutri-Score categories between both versions, Chi-square tests were conducted. This test was suitable to assess whether there were significant differences in the categorical classification distribution (A to E) before and after the update.

To evaluate Nutri-Score’s ability to discriminate nutritional quality across beverages, the number of colors available per category was analyzed. Finally, the agreement between the 2015 and 2023 classifications was evaluated using the kappa index, which is suitable for measuring concordance between categorical assessments beyond chance.

For all statistical tests, *p* < 0.05 was considered statistically significant. All analyses were performed using Stata/SE (version 14.0, StataCorp, College Station, TX, USA).

## 3. Results

### 3.1. Characteristics of the Product Sample

Table 1 presents the nutritional characteristics of the different beverage categories. Among them, plant-based beverages had the highest fiber content, and a higher median energy content compared to the other categories. Legume-based beverages and milk had the highest protein content.

### 3.2. Comparison of Original and Updated NS-NPM Scores and Nutri-Score Categories Across Beverage Types

Table 2 compares NS-NPM scores between the original and updated versions across beverage categories. The NS-NPM score using the original algorithm ranged from −1 for legume beverages to 11 for sugar-sweetened beverages. In contrast, the updated 2023 NS-NPM version showed a broader range, from −2 points for legume-based beverages to 10 points for sugar-sweetened beverages.

The relative variation in NS-NPM scores between the 2015 and 2023 versions differed significantly across beverage categories. The largest increase was observed in plant-based beverages mix (+733%), cereal-based beverages (+651.3%), nut beverages (+343.9%), artificially sweetened beverages (+190.3%), and milkshakes (+98.9%). In contrast, legume-based beverages (−1.4%), sugar-sweetened beverages (−4.2%), fruit juices (−12.7%), vegetable juices (+12.3%), and fruit juice concentrates and nectars (+18%) showed smaller variations in score.

**Figure 2:**
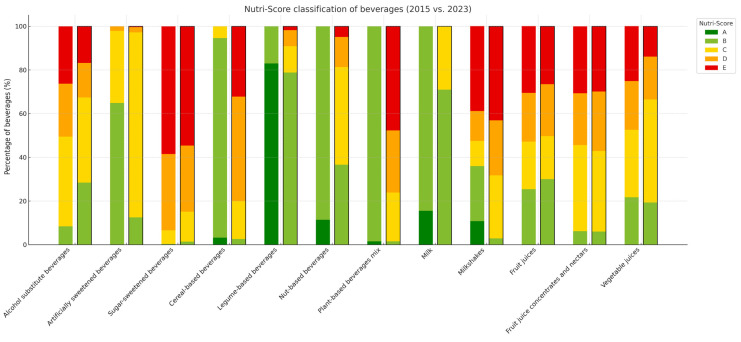
Beverage classification across Nutri-Score versions. For each beverage category, the left bar corresponds to the 2015 algorithm and the right bar to the 2023 algorithm (outlined in black).

Figure 2 presents the proportion of products classified under each Nutri-Score version and the level of agreement between the 2015 and 2023 classifications. Legume-based beverages, milk, and milkshakes were no longer classified as A in the updated version. While 83% of legume-based beverages received an A in the original version, most were reclassified to B under the updated Nutri-Score. As for milk and milkshakes, while 15.5% and 10.8% of them, respectively, received an A in the original version, they were reclassified to B or lower in the revised Nutri-Score.

Compared to the original Nutri-Score algorithm classification, the updated version resulted in a 6 percentage points increase in beverages classified as D, and a 14.4 percentage points decrease in those classified as B, and an 8.6 percentage points decrease in those classified as C. Detailed information is shown in Supplemental Table S1.

The highest agreement (Kappa index) between original and updated Nutri-Score classifications was observed for fruit juice concentrates and nectars (82.1%), fruit juices (77.7%), and sugar-sweetened beverages (65.9%). In contrast, legume-based beverages showed the lowest agreement (1.2%) (Supplemental Table S1).

**Figure 3:**
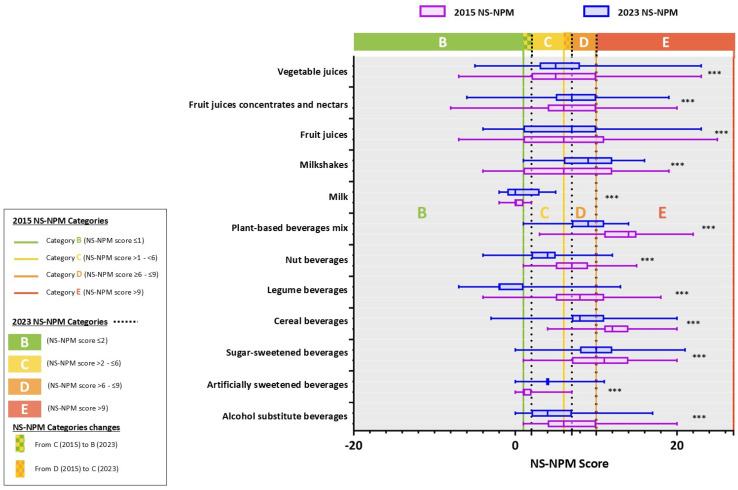
Boxplot of the distribution of 2015 and 2023 NS-NPM scores and Nutri-Score categories across beverage types. Abbreviations: NS-NPM: Nutri-Score Nutrient Profiling Model System. Asterisks indicate comparisons between Nutri-Score categories in the 2015 and 2023 versions, analyzed using the Chi-Square test with the statistical significance level: *** *p* < 0.001. The boxplot interpretation is as follows: the boundary closest to the right indicates the 25th percentile, the line within the box marks the median, and the boundary furthest to the right indicates the 75th percentile. Nutri-Score categories are displayed at the top of the figure. Cut-offs for the 2015 NS-NPM categories are represented within the plot area by colored vertical lines (―), while cut-offs for the 2023 NS-NPM categories are represented using black dotted vertical lines (· · ·).

Figure 3 illustrates the classification differences between the two Nutri-Score versions. The boxplot shows that most beverages were assigned to different categories in the updated version compared to the original. Milk products, which were predominantly classified as B in the original version, were now distributed between B and C. Similarly, while most plant-based beverages, previously classified as B, were reassigned between categories B and C in the updated version. Fruit juices, initially spread across categories C and E, were reclassified between B and D.

## 4. Discussion

To the best of our knowledge, this is the first study assessing the impact of the 2023 NS-NPM update on beverage classification in Spain. Although the Scientific Committee of Nutri-Score has addressed beverage classification in its revision process and previous studies have assessed its impact in other countries—such as Sarda et al. in France [18]—our study provides the first country-specific analysis for the Spanish market.

Compared to the original Nutri-Score, the updated algorithm downgraded artificially sweetened beverages due to penalties for non-nutritive sweeteners and reclassified milkshakes based on sugar content.

### 4.1. New Product Reclassification

Our results indicate that the updated Nutri-Score algorithm altered the categorical ranking for most beverage types, except for sugar-sweetened beverages, legume-based beverages, fruit juices, and fruit juice concentrates and nectars, which remained largely unchanged. The lowest agreement between the original and updated versions was observed in plant-based beverages (particularly legume-based beverages), alcohol substitutes, artificially sweetened beverages, and milkshakes. Conversely, the highest agreement was observed for fruit juices, fruit juice concentrates and nectars, vegetable juices, and sugar-sweetened beverages.

The most significant classification shifts occurred for cereal-, nut-, and plant-based beverage mixes, milkshakes, and artificially sweetened beverages, which received notably higher score under the updated NS-NPM algorithm and, consequently, a less favorable ranking. This shift is primarily due to two major changes introduced in the 2023 Nutri-Score algorithm. First, plant-based beverages, milk, and milkshakes are now beverages rather than regular foods, which subjects them to stricter penalties for sugar and energy content. Second, the updated algorithm places greater emphasis on positive components such as protein.

Many nut- and cereal-based plant beverages are relatively low in protein and high in sugar, which limits their ability to gain favorable points while increasing their penalties under the new scoring system. As a result, these beverages were heavily downgraded, reflecting their nutrient composition more accurately than under the previous model [18,30].

In contrast, legume-based beverages—such as soy and pea drinks—typically have higher protein content and lower levels of added sugars. This allowed them to maintain their classifications as the updated Nutri-Score algorithm differentiates them from other plant-based beverages by prioritizing higher protein content. This adjustment aligns with previous research highlighting the importance of protein and fiber cut-off points in Nutri-Score classification [17,18,31]. By refining these criteria, the new Nutri-Score enables a clearer distinction among different plant-based beverages, shedding light on their actual nutritional composition, offering consumers more accurate and meaningful guidance when comparing alternatives within a balanced diet. These findings are consistent with studies conducted in France and Germany [16,18,31], further supporting the Nutri-Score’s role in improving discrimination regarding beverage nutritional quality.

Categorization of sugar-sweetened beverages, fruit juices, fruit juice concentrates and nectars, and vegetable juices saw minimal changes as the updated algorithm maintained similar classification criteria for these high-sugar products. Meanwhile, artificially sweetened beverages experienced a sharp increase in their score after non-nutritive sweeteners were penalized in the revised scoring as an unfavorable component [18,30]. This increase in scoring led to their reclassification from Nutri-Score B to C. Similarly, cereal beverages, nut beverages, plant-based beverages mix, milkshakes saw a substantial increase, reflecting their new classification as beverages and the stricter penalties for sugar content. In contrast, legume-based beverages showed minimal score variation, which resulted in the maintenance of their Nutri-Score classification primarily shifting from A to B. This aligns with the revised algorithm prioritizing higher protein content in plant-based beverages.

### 4.2. Consumer Information and Public Health

The findings of this study suggest important implications for how Nutri-Score may influence consumer awareness of beverage nutritional quality. The growing body of evidence on Nutri-Score’s efficacy across European countries demonstrates its role in guiding consumer choices and supporting public health efforts [8]. The reclassification of certain plant-based beverages (except legume-based) provides consumers with more accurate information about their actual nutritional composition, particularly regarding sugars and protein content. This enhanced discrimination helps clarify that not all plant-based beverages are nutritionally equivalent, aligning consumer expectations with their real dietary contributions.

By reclassifying artificially sweetened beverages and milkshakes, the updated Nutri-Score may contribute to reshaping consumer perceptions of these products. Downgrading artificially sweetened beverages reflects emerging evidence of potential health risks from non-nutritive sweeteners [30,31]. This change may help raise awareness and challenge the widespread perception of these products as healthier alternatives.

Taken together, the differential impact of the Nutri-Score update across beverage types may have important implications for consumer behavior. Categories such as artificially sweetened beverages, cereal- and nut-based drinks, and milkshakes were most affected, receiving less favorable classifications due to high sugar content, low protein, or the presence of non-nutritive sweeteners. This may shift consumer preferences toward unsweetened dairy or legume-based plant alternatives, which maintained more favorable scores. In contrast, the minimal changes observed in fruit juices and sugar-sweetened beverages may limit the update’s impact in these already penalized categories, unless further algorithm refinements are introduced. From a public health perspective, the revised Nutri-Score provides stronger incentives for reformulation in certain beverage groups and clearer information for consumers seeking healthier options, reinforcing its role as a strategic tool for dietary improvement.

While the updated algorithm led to statistically significant changes in NS-NPM scores, their practical implications differ depending on the beverage category. For instance, the observed downgrading of artificially sweetened beverages and plant-based beverages may influence consumer perceptions, potentially discouraging their consumption or prompting reformulation to improve nutritional profiles. In contrast, the relatively minor changes in the classification of fruit juices and sugar-sweetened beverages suggest limited added impact in categories already penalized under the original model. Overall, the updated Nutri-Score algorithm appears to enhance the discriminatory capacity in specific beverage groups, aligning the label more closely with public health goals, though the extent of practical impact will depend on consumer response and industry adaptation.

The minimal impact of the Nutri-Score update on fruit juices and sugar-sweetened beverages suggests that these products continue to be classified in less favorable Nutri-Score categories. However, it remains unclear whether the algorithm effectively differentiates between naturally occurring and added sugars in fruit juices. This limitation reinforces previous concerns that fruit juices may not be adequately penalized, despite their high free sugar content [9], and raises questions about whether consumers continue to perceive them as a healthier alternative to sugar-sweetened beverages, despite their similar sugar content. Given the declaration of added sugar is not currently mandatory on the food label, the current Nutri-Score algorithm is based on total sugar content and does not distinguish between intrinsic and added or free sugars. Incorporating this differentiation in the mandatory nutritional labeling and future updates could better reflect the health risks associated with sugary beverages. This would be particularly relevant from a public health perspective as fruit juices often contain high amounts of free sugars while being perceived as healthy. Improving this aspect of the algorithm could enhance consumer understanding and ensure greater alignment with dietary guidelines that aim to reduce free sugar intake. Analyzing products using FOP labeling systems like Nutri-Score enables consumers to quickly and easily assess the nutritional quality of beverages [32]. The updated 2023 NS-NPM algorithm enhances discrimination in product classification, potentially encouraging healthier consumer choices, such as opting for less sugary milkshakes or favoring plant-based beverages rich in protein and low in sugar. While this labeling strategy contributes to public health efforts, additional research is necessary to determine its long-term influence on dietary habits across different socioeconomic groups [9,32]. By providing a clearer understanding of beverages’ nutritional quality, this study reinforces the importance of FOP labeling in guiding consumer choices. Additionally, it underscores the role of Nutri-Score in promoting discrimination and encouraging healthier product selection. The findings reinforce the need for clear communication strategies to ensure that consumers correctly interpret the Nutri-Score classifications and their implications for health.

### 4.3. Impact on the Food Industry

Food policy plays a key role in driving product reformulation within the food industry, but consumer preferences also shape market supply [33]. For consumers to influence reformulation effectively, they need clear and accessible nutritional information, such as that provided by Nutri-Score labeling [32].

The updated Nutri-Score algorithm may encourage product reformulation across multiple beverage categories. The downgrading of artificially sweetened beverages could push manufacturers to reduce the use of non-nutritive sweeteners, particularly as consumer awareness of sweetener-related health concerns increases. Similarly, manufacturers of smoothies, as well as cereal-, nut- and plant-based beverages, may seek to reduce sugar content to improve their Nutri-Score rating, aligning with public health efforts to reduce sugar consumption. Additionally, producers of plant-based beverages may also aim to increase protein content to achieve a more favorable classification under the updated Nutri-Score algorithm. This study highlights that the updated Nutri-Score algorithm provides a more precise assessment of sugar content in milkshakes and plant-based beverages, and the presence of non-nutritive sweeteners in artificially sweetened beverages, reinforcing the potential for industry-led adjustments.

Conversely, the improved classification of legume-based beverages may incentivize greater market availability of these plant-based options. This shift aligns with dietary guidelines promoting plant-based diets, particularly given their higher protein and fiber content [17]. The favorable reclassification of plant-based beverages could also drive reformulation efforts in this category, particularly to enhance protein content further.

Evidence suggests that implementing FOP labeling encourages industry responses, with interpretative systems like Nutri-Score proving particularly effective in promoting product reformulation [34]. As consumer awareness of Nutri-Score classifications grows, manufacturers may adjust beverage formulations to improve their scores, reinforcing the role of labeling policies in shaping healthier food environments.

The updated Nutri-Score algorithm may also shift consumer behavior by increasing the visibility of nutritional differences between beverage subtypes, such as between sweetened and unsweetened plant-based drinks or between dairy and plant alternatives with varying protein content levels. Over time, this could influence purchasing decisions, favoring products with more favorable labels. In parallel, food and beverage manufacturers may be incentivized to reformulate products not only to avoid negative classification but also to maintain market competitiveness. These dual dynamics, consumer responsiveness and industry adaptation, are key to understanding the real-world effectiveness of FOP labeling systems like Nutri-Score in improving population diets.

### 4.4. Food Labeling Policy

The World Health Organization has urged its member states, particularly in Europe, to adopt comprehensive regulations and policies, including the implementation of FOP labeling systems [8]. Nutri-Score is a validated FOP labeling system designed to enhance consumer awareness and support public health initiatives by providing a standardized approach to marketing targeted at children, influencing school nutrition programs, and informing taxation policies on unhealthy foods [35,36].

As demonstrated in this study, the use of FOP labeling algorithms, such as Nutri-Score, reinforces their value in guiding consumers toward healthier choices. Although this study focused specifically on beverages available in Spain, the findings support the broader relevance of validated FOP systems in public health strategies. Nutri-Score stands out for its interpretability and consumer preference, and its underlying nutrient profiling model has been extensively validated across multiple studies and systematic reviews [37]. Further research is warranted to assess how such systems perform across diverse food categories and national contexts.

Among the labeling systems evaluated in 2020, Nutri-Score was found to meet key criteria, including efficacy, consumer appeal, and simplicity. For these reasons, it remains the most suitable candidate for widespread adoption across the EU to improve public health and reduce obesity rates [38]. However, its implementation remains uneven, with some countries fully adopting it (e.g., France, Belgium, Germany, and Spain), while others (e.g., Italy) oppose its use, citing concerns over its impact on traditional food products [39]. Additionally, harmonization efforts require addressing potential legal and trade implications, particularly regarding mandatory vs. voluntary labeling policies. Further research and policy discussions are needed to overcome these challenges and ensure that Nutri-Score achieves its intended public health benefits across diverse food markets.

Although this study focused on the Spanish market, its findings may also be relevant in other countries with similar beverage availability and labeling regulations. However, variations in dietary patterns, consumer preferences, and the penetration of plant-based or artificially sweetened beverages may influence how the Nutri-Score update impacts product classification and consumer choices in different contexts. Future comparative studies could explore how the updated algorithm performs across countries with diverse food cultures, informing harmonized approaches to FOP labeling in Europe and beyond.

### 4.5. Policy Implications

The findings highlight the potential public health impact of the updated Nutri-Score. The revision provides clearer guidance for consumers, particularly in identifying low-protein plant-based beverages, high-sugar milkshakes, and artificial sweetened beverages. Through improved discrimination within and across beverage types, the updated algorithm reinforces the role of Nutri-Score as an effective FOP labeling system.

Based on these findings, several policy-oriented recommendations may be considered to enhance the relevance and effectiveness of Nutri-Score. First, incorporating a distinction between intrinsic and added or free sugars—particularly in beverages like fruit juices—could improve the algorithm’s alignment with public health recommendations. Second, adjusting the algorithm to better reward positive components such as protein may help distinguish nutritionally richer plant-based alternatives from those with lower nutritional value.

In addition, consumer education strategies could play a key role in improving the understanding and appropriate use of the label, especially for products where perceived healthfulness does not align with Nutri-Score classification. For industry stakeholders, the updated algorithm offers incentives to reformulate beverages by reducing added sugar and increasing beneficial nutrients. Health professionals and educators can also use Nutri-Score to guide nutritional counseling and support informed consumer decision-making. While Nutri-Score continues to evolve, these complementary efforts may help maximize its impact in promoting healthier beverage choices.

Looking ahead, the findings of this study underscore the importance of continuously evolving FOP labeling systems to reflect advances in nutritional science and public health priorities. As beverage markets diversify and consumer awareness increases, evidence-based tools like Nutri-Score may contribute to shaping healthier food environments. Policymakers, researchers, and industry stakeholders should continue collaborating to ensure that labeling systems remain effective, equitable, and responsive to emerging dietary challenges.

### 4.6. Strengths and Limitations

This study has several strengths. First, it is the first study to compare the 2023 NS-NPM algorithm with the original version for beverages marketed in Spain. Second, it includes a large and diverse sample of beverages, drawn from the “Drink Base” database [19], a comprehensive and unique resource on beverages available in the Spanish market.

However, some limitations must be acknowledged. We cannot ensure all beverages available in Spain were included in the analysis, and some product formulations in the “Drink Base” database [19] may have changed since data collection. The sample was limited to beverages available in major supermarket chains, which may introduce selection bias by excluding products from smaller or regional retailers. Another limitation is the reliance on nutritional labeling, which may introduce potential bias.

Moreover, the study did not account for product reformulations that may have occurred after data collection, nor did it account for seasonal or regional variations in product availability. These factors may limit the generalizability of the findings to the full range of beverages currently available on the Spanish market. Furthermore, the study assumes zero fiber content for products where this information is missing as fiber declaration is not mandatory under European regulations [27]. This approach, while necessary, may have led to an underestimation of fiber content.

Another important limitation is the lack of information regarding added or free sugars in the labeling data used due to lack of mandatory declaration in UE. Since the current Nutri-Score algorithm does not differentiate between intrinsic and added sugars, the nutritional assessment of certain beverages, especially fruit juices, may be limited. Future research should explore mechanisms to estimate or directly include added sugar content in profiling algorithms.

Additionally, future studies should explore consumer responses to the updated Nutri-Score, especially in purchasing behavior and dietary choices, as well as potential adjustments in beverage formulations by manufacturers. Understanding how these changes influence consumer behavior and industry reformulation will be crucial in maximizing the effectiveness of FOP labeling systems. Future research could also include longitudinal studies tracking actual purchasing behavior before and after Nutri-Score implementation, experimental studies assessing consumer comprehension and the use of the label, and cross-national comparisons to evaluate the impact of Nutri-Score in different policies and market contexts. These approaches would provide stronger evidence on the real-world effectiveness of the updated algorithm and inform future refinements of FOP labeling systems.

Finally, although this study is focused on the Spanish market, differences in dietary habits, beverage consumption patterns, and FOP labeling regulations across countries may limit the transferability of the findings to other populations. Future research is needed to assess how the updated Nutri-Score algorithm performs in different cultural, nutritional, and regulatory contexts, which could support the development of harmonized public health strategies across Europe and beyond.

## 5. Conclusions

This study is the first to evaluate the impact of the 2023 NS-NPM algorithm update on the classification of non-alcoholic beverages in the Spanish market. The updated model improves beverage classification by more accurately distinguishing between sugar-sweetened milkshakes and artificially sweetened products. It strengthens the public health relevance of Nutri-Score by refining how these beverages are categorized, providing clearer guidance to consumers and reinforcing its credibility as a FOP labeling tool.

However, while the revised algorithm improves overall discrimination, the classification of plant-based beverages remains complex. Several categories were significantly downgraded due to their sugar, protein, and energy content. Moreover, fruit juices and sugar-sweetened beverages largely retained their previous classification, which raises concerns about whether Nutri-Score adequately accounts for the free sugar content of these beverages or distinguishes between naturally occurring and added sugars.

These findings have direct implications for food industry reformulation strategies, particularly for beverages containing non-nutritive sweeteners and milkshakes and plant-based beverages with high sugar content or low protein density. The Nutri-Score update may encourage manufacturers to reduce added sugars or increase protein content in plant-based beverages to achieve more favorable classifications.

As Nutri-Score continues to evolve, its potential to shape consumer choices and influence industry practices makes it a key public health instrument. Future research should explore refinements to better align the algorithm with dietary recommendations and assess its real-world impact through longitudinal and cross-national studies.

This study contributes novel, context-specific evidence that can inform both national policies and the broader harmonization of FOP labeling systems in Europe and beyond. Its findings may support health authorities in evaluating and refining nutrition labeling regulations, guide manufacturers in product development and reformulation strategies, and assist public health practitioners and educators in developing targeted interventions to improve beverage choices among consumers.

## Figures and Tables

**Table 1:** General characteristics of beverage categories based on NS-NPM components.

Type of Beverage Product (100 mL)	Energy (kcal)	Sugar (g)	Total Fat (g)	SFA (g)	Salt (g)	Protein (g)	Fiber (g)	F, V, L, N, and OO, RO, and WO (%)
Alcohol-substitute beverages (*n* = 95)	22.0 [0.95; 72.0]	2.1 [0.0; 18.0]	0.0 [0.0; 0.5]	0.0 [0.0; 0.1]	0.01 [0; 0.3]	0.1 [0.0; 1.0]	0.0 [0.0; 3.0]	0.0 [0.0; 24.0]
Artificially sweetened beverages (*n* = 288)	1.0 [0.0; 16.0]	0.0 [0.0; 9.0]	0.0 [0.0; 0.9]	0.0 [0.0; 0.9]	0.02 [0; 0.24]	0 [0.0; 1.5]	0.0 [0.0; 0.6]	0.0 [0.0; 35.0]
Sugar-sweetened beverages (*n* = 648)	33.0 [0.25; 106.0]	7.6 [0.0; 16.0]	0.0 [0.0; 1.2]	0.0 [0.0; 0.5]	0.01 [0.0; 1.0]	0 [0.0; 1.17]	0.0 [0.0; 0.7]	0.0 [0.0; 100.0]
Cereal-based beverages (*n* = 313)	52.0 [22.0; 106.0]	6.1 [0.0; 15.2]	1.1 [0.0; 12.0]	0.2 [0.0; 1.0]	0.09 [0.0; 0.55]	0.6 [0.0; 3.6]	0.4 [0.0; 2.7]	0.0 [0.0; 29.0]
Legume-based beverages (*n* = 165)	43.0 [25.0; 79.0]	2.9 [0.0; 12.0]	1.8 [0.5; 2.9]	0.3 [0.0; 0.27]	0.49 [0.0; 2.9]	3.1 [1.5; 5.0]	0.49 [0.0; 2.9]	11.0 [0.0; 97.4]
Nut-based beverages (*n* = 123)	33.0 [10.42; 85.0]	2.5 [0.0; 8.0]	1.9 [0.2; 5.0]	0.2 [0.0; 0.9]	0.1 [0.0; 0.19]	0.7 [0.0; 4.2]	0.3 [0.0; 2.2]	2.8 [0.0; 96.9]
Plant-based beverages mix (*n* = 67)	60.0 [14.58; 83.0]	5.6 [0.0; 9.0]	1.5 [0.4; 4.9]	0.4 [0.07; 2.6]	0.1 [0.0; 0.3]	0.5 [0.0; 2.9]	0.5 [0.1; 1.3]	0.02 [0.0; 15.0]
Milk (*n* = 148)	46.0 [31.0; 70.8]	4.7 [4.4; 7.4]	1.6 [0.0; 3.7]	1.05 [0.0; 2.5]	0.13 [0.1; 0.2]	3.1 [3.0; 5.4]	0.0 [0.0; 0.0]	0.0 [0.0; 0.0]
Milkshakes (*n* = 139)	45.0 [11.0; 88.0]	7.5 [1.9; 13.0]	0.1 [0.0; 2.8]	0.0 [0.0; 1.8]	0.1 [0.0; 0.25]	0.5 [0.0; 8.3]	0.0 [0.0; 1.2]	3.2 [0.0; 90.0]
Fruit juices (*n* = 193)	48.0 [12.0; 91.0]	9.2 [0.0; 15.0]	0.2 [0.0; 6.0]	0.03 [0.0; 4.7]	0.02 [0.0; 7.0]	0.5 [0.0; 2.6]	0.0 [0.0; 11.0]	99.0 [0.0; 100.0]
Fruit juice concentrates and nectars (*n* = 916)	45.0 [4.0; 95.0]	9.9 [0.0; 23.0]	0.1 [0.0; 2.0]	0.0 [0.0; 1.5]	0.01 [0.0; 0.4]	0.3 [0.0; 2.9]	0.0 [0.0; 2.5]	99.0 [0.0; 100.0]
Vegetable juices (*n* = 337)	40.0 [2.4; 186.0]	4.1 [0.0; 19.0]	0.1 [0.0; 14.0]	0.0 [0.0; 2.2]	0.05 [0.0; 2.0]	0.6 [0.0; 3.5]	0.0 [0.0; 3.4]	54.0 [0.0; 100.0]

Data are expressed as median [min;max]. Abbreviations: SFA, Saturated Fatty Acids; F, Fruits; V, Vegetables; L, Legumes; N, Nuts; OO, Olive oil; RO, Rapeseed oil; WO, Walnut oil.

**Table 2:** Comparison of original and updated NS-NPM scores and Nutri-Score categories across beverage types.

Type of Beverage	2015 NS-NPM	2023 NS-NPM	Relative Variation (%)	*p*-Value *	Spearman Coefficient **	2015 Nutri-Score Categories	2023 Nutri-Score Categories
Alcohol-substitute beverages (*n* = 95)	6.0 [1.0; 20.0]	4.0 [0.0; 17.0]	−8.2	<0.001	0.8	D	C
Artificially sweetened beverages (*n* = 288)	1.0 [0.0; 7.0]	4.0 [0.0; 11.0]	190.3	<0.001	0.5	B	C
Sugar-sweetened beverages (*n* = 648)	11.0 [1.0; 20.0]	10.0 [0.0; 21.0]	−4.2	<0.001	0.8	E	E
Cereal-based beverages (*n* = 313)	1.0 [−2.0; 4.0]	8.0 [−3.0; 20.0]	651.3	<0.001	0.7	B	D
Legume-based beverages (*n* = 165)	−1.0 [−6.0; 1.0]	−2.0 [−7.0; 13.0]	−1.4	0.010	0.6	A	B
Nut-based beverages (*n* = 123)	0.0 [−5.0; 1.0]	4.0 [−4.0; 12.0]	343.9	<0.001	0.6	B	C
Plant-based beverages mix (*n* = 67)	1.0 [−1.0; 2.0]	9.0 [1.0; 14.0]	733.0	<0.001	0.6	B	D
Milk (*n* = 148)	0.0 [−2.0; 2.0]	0.0 [−2.0; 5.0]	36.8	0.830	0.7	B	B
Milkshakes (*n* = 139)	6.0 [−4.0; 19.0]	9.0 [1.0; 16.0]	98.9	<0.001	0.7	C	D
Fruit juices (*n* = 193)	6.0 [−7.0; 25.0]	7.0 [−4.0; 23.0]	−12.7	0.760	0.9	D	D
Fruit juice concentrates and nectars (*n* = 916)	6.0 [−8.0; 20.0]	7.0 [−6.0; 19.0]	18.0	<0.001	0.9	D	D
Vegetable juices (*n* = 337)	5.0 [−7.0; 23.0]	5.0 [−5.0; 23.0]	12.3	0.006	0.9	C	C

Abbreviations: NS-NPM: Nutri-Score Nutrient Profiling Model. Data are expressed as median [min;max]. Relative variation: ((2023 NS-NPM score − 2015 NS-NPM score)/2015 NS-NPM score) × 100. Nutri-Score Categories: A (dark green): Highest nutritional quality. B (light green): Good nutritional quality. C (yellow): Moderate nutritional quality. D (orange): Low nutritional quality. E (red): Lowest nutritional quality. * Wilcoxon signed-rank test was used to determine differences between the 2015 and 2023 NS-NPM scores. ** Spearman correlation coefficient between the 2015 and 2023 NS-NPM scores.

## Data Availability

The raw data supporting the conclusions of this article will be made available by the authors on request due to privacy.

## Supplementary Materials

The following supporting information can be downloaded at https://www.mdpi.com/article/10.3390/nu17091521/s1. Supplemental Figure S1: Attribution of points based on nutrient content and other elements per 100 mL of beverage in the 2015 NS-NPM. Supplemental Figure S2: Attribution of points based on nutritional content and other elements per 100 g or 100 mL of plant-based beverages, milk, and milkshakes in the 2015 NS-NPM. Supplemental Figure S3: Attribution of points based on nutritional content and other elements per 100 mL of beverage in the 2023 NS-NPM. Supplemental Table S1: Percentage of beverage classified by Nutri-Score categories in the original and updated versions and agreement between both versions.

## Abbreviations

The following abbreviations are used in this manuscript:

NS-NPM	Nutri-Score Nutrient profiling Model
FOP	Front-of-pack
