# Supplementary material for: The Nutritional Profile of Spanish Beverages: A Comparative Evaluation of the Original and Updated Nutri-Score Algorithm
Source: Nutrients. 2025 Apr 30;17(9):1521. doi: 10.3390/nu17091521 (PMC12073336; doi:10.3390/nu17091521)
Supplement: Supplementary file 1 [file nutrients-17-01521-s001.zip › nutrients-3592134-supplementary.pdf]

Supplemental figure S1. Attribution of points based on nutrient content and other elements per 100ml of beverage in the 2015 NS-NPM.

**Unfavorable components – Points N**				
**Points**	Energy (kJ) (a)	Sugars (g) (b)	SFA (g) (c)	Sodium (mg) (d)
**0**	≤0	≤0	≤1	≤90
**1**	≤30	≤1.5	>1	>90
**2**	≤60	≤3	>2	>180
**3**	≤90	≤4.5	>3	>270
**4**	≤120	≤6	>4	>360
**5**	≤150	≤7.5	>5	>450
**6**	≤180	≤9	>6	>540
**7**	≤210	≤10.5	>7	>630
**8**	≤240	≤12	>8	>720
**9**	≤270	≤13.5	>9	>810
**10**	>270	>13.5	>10	>900
**Total**	**Points N= (a) + (b) + (c) + (d)**			

**Favorable components – Points P**			
**Points**	Fruit, vegetables and legumes (%) (a)	Fiber (g) (b)	Protein (g) (c)
**0**	≤40	≤0.7	≤1.6
**1**		>0.7	>1.6
**2**	>40	>1.4	>3.2
**3**		>2.1	>4.8
**4**	>60	>2.8	>6.4
**5**		>3.5	>8.0
**6**			
**7**			
**8**			
**9**			
**10**	>80		
**Total**	**Points P= (a) + (b) + (c)**		

Final score= Points N – Points P

Final Nutri-Score thresholds

**2015 NS-NPM score**	**Category**	**Color**
Water	A	Dark green
<1 to 1	B	Light green
>1 to 5	C	Yellow
>5 to 9	D	Light Orange
10 to >10	E	Dark Orange

Supplemental figure S2. Attribution of points based on nutritional content and other elements per 100g or 100ml of plant-based beverages, milk and milkshakes in the 2015 NS-NPM.

Unfavorable components – Points N				
**Points**	Energy (kJ) (a)	Sugars (g) (b)	SFA (g) (c)	Sodium (mg) (d)
**0**	≤335	≤4.5	≤1	≤90
**1**	>335	>4.5	>1	>90
**2**	>670	>9	>2	>180
**3**	>1005	>13.5	>3	>270
**4**	>1340	>18	>4	>360
**5**	>1675	>22.5	>5	>450
**6**	>2010	>27	>6	>540
**7**	>2345	>31	>7	>630
**8**	>2680	>36	>8	>720
**9**	>3015	>40	>9	>810
**10**	>3350	>45	>10	>900
**Total**	**Points N= (a) + (b) + (c) + (d)**			

Favorable components – Points P			
**Points**	Fruit, vegetables and legumes (%) (a)	Fiber (g) (b)	Protein (g) (c)
**0**	≤40	≤0.7	≤1.6
**1**	>40	>0.7	>1.6
**2**	>60	>1.4	>3.2
**3**	-	>2.1	>4.8
**4**	-	>2.8	>6.4
**5**	>80	>3.5	>8.0
**6**			
**7**			
**8**			
**9**			
**10**			
**Total**	**Points P= (a) + (b) + (c)**		

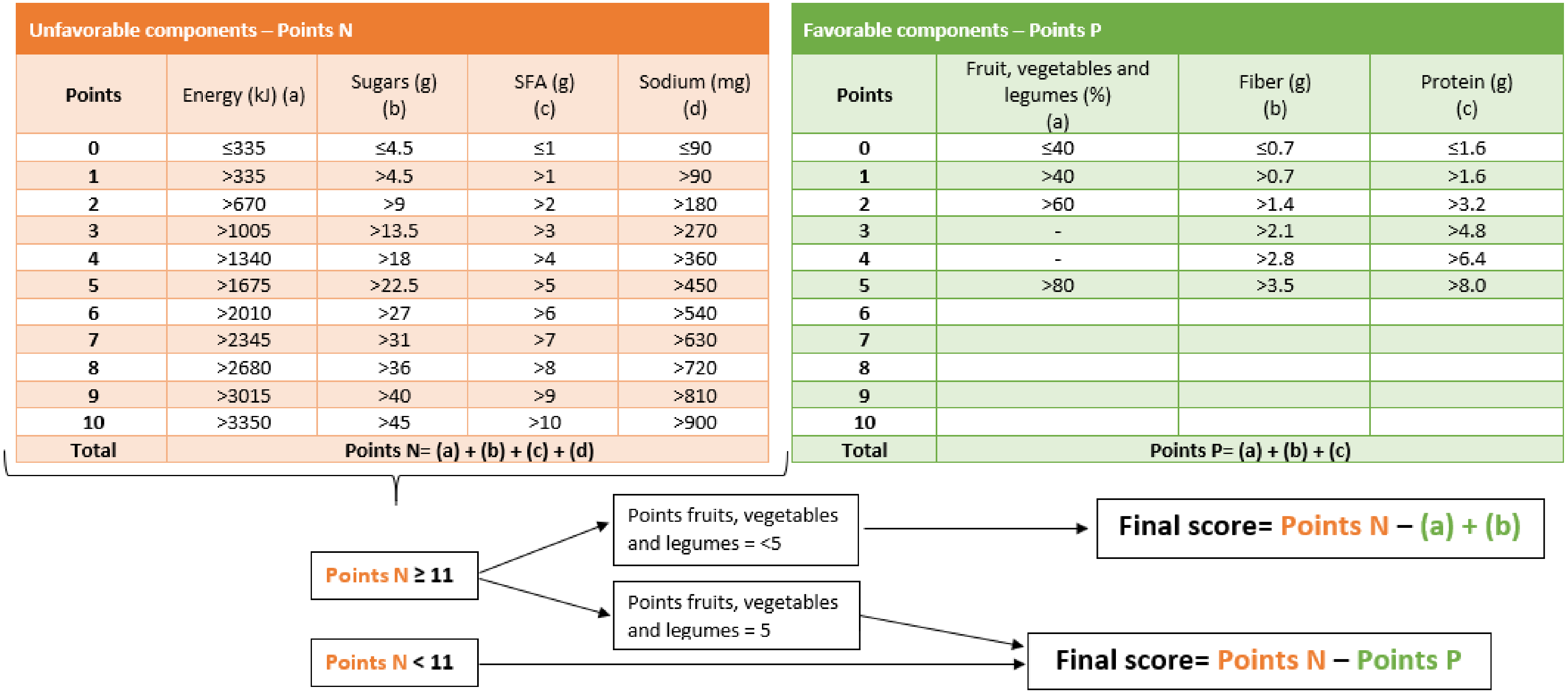

Final Nutri-Score thresholds

2015 NS-NPM score	Category	Color
≤-1 to -1	A	Dark green
0 to 2	B	Light green
3 to 10	C	Yellow
11 to 18	D	Light Orange
19 to >19	E	Dark Orange

Supplemental figure S3. Attribution of points based on nutritional content and other elements per 100ml of beverage in the 2023 NS-NPM.

Unfavorable components – Points N

Points	Energy (kJ) (a)	Sugars (g) (b)	SFA (g) (c)	Salt (g) (d)	Non-nutritive sweeteners (Presence/absence) (e)
0	≤30	≤0.5	≤1	≤0.2	
1	≤90	≤2	>1	>0.2	
2	≤150	≤3.5	>2	>0.4	
3	≤210	≤5	>3	>0.6	
4	≤240	≤6	>4	>0.8	Presence
5	≤270	≤7	>5	>1	
6	≤300	≤8	>6	>1.2	
7	≤330	≤9	>7	>1.4	
8	≤360	≤10	>8	>1.6	
9	≤390	≤11	>9	>1.8	
10	>390	>11	>10	>2	
11				>2.2	
12				>2.4	
13				>2.6	
14				>2.8	
15				>3	
16				>3.2	
17				>3.4	
18				>3.6	
19				>3.8	
20				>4	
**Total**	**Points N= (a) + (b) + (c) + (d) + (e)**				

Favorable components – Points P

Points	Fruit, vegetables and legumes (%) (a)	Fiber (g) (b)	Protein (g) (c)
0	≤40	≤3	≤1.2
1	-	>3	>1.2
2	>40	>4.1	>1.5
3	-	>5.2	>1.8
4	>60	>6.3	>2.1
5	-	>7.4	>2.4
6	>80		>2.7
7			>3.0
**Total**	**Points P= (a) + (b) + (c)**		

Final score= Points N – Points P

Final Nutri-Score thresholds

2023 NS-NPM score	Category	Color
Water	A	Dark green
<1 to 1	B	Light green
>1 to 5	C	Yellow
>5 to 9	D	Light Orange
10 to >10	E	Dark Orange

Supplemental table S1. Percentage of beverage classified by Nutri-Score categories in the original and updated versions and agreement between both versions.

Type of beverage	Version of NS-NPM	Nutri-Score categories A % (n)	B % (n)	C % (n)	D % (n)	E % (n)	Agreement (Kappa index)
Alcohol substitute beverages (n= 95)	Original	0.0 (0)	8.4 (8)	41.1 (39)	24.2 (23)	26.3 (25)	31.6
	Updated	0.0 (0)	28.4 (27)	39.0 (37)	15.8 (15)	16.8 (16)	
Artificially sweetened beverages (n= 288)	Original	0.0 (0)	64.9 (187)	33.0 (95)	2.1 (6)	0.0 (0)	41.7
	Updated	0.0 (0)	12.5 (36)	84.7 (244)	2.4 (7)	0.4 (1)	
Sugar-sweetened beverages (n= 648)	Original	0.0 (0)	0.3 (2)	6.3 (41)	34.9 (226)	58.5 (379)	65.9
	Updated	0.0 (0)	1.4 (9)	13.7 (89)	30.3 (196)	54.6 (354)	
Cereal-based beverages (n= 313)	Original	3.2 (10)	91.4 (286)	5.4 (17)	0.0 (0)	0.0 (0)	2.2
	Updated	0.0 (0)	2.6 (8)	17.3 (54)	47.9 (150)	32.3 (101)	
Legume-based beverages (n= 165)	Original	83.0 (137)	17.0 (28)	0.0 (0)	0.0 (0)	0.0 (0)	1.2
	Updated	0.0 (0)	78.8 (130)	12.1 (20)	7.3 (12)	1.8 (3)	
Nut-based beverages (n= 123)	Original	11.4 (14)	88.6 (109)	0.0 (0)	0.0 (0)	0.0 (0)	26.8
	Updated	0.0 (0)	36.6 (45)	44.7 (55)	13.8 (17)	4.9 (6)	
Plant-based beverages mix (n= 67)	Original	1.5 (1)	98.5 (66)	0.0 (0)	0.0 (0)	0.0 (0)	1.5
	Updated	0.0 (0)	1.5 (1)	22.4 (15)	28.4 (19)	47.8 (32)	
Milk (n= 148)	Original	15.5 (23)	84.5 (125)	0.0 (0)	0.0 (0)	0.0 (0)	55.4
	Updated	0.0 (0)	71.0 (105)	29.0 (43)	0.0 (0)	0.0 (0)	
Milkshakes (n= 139)	Original	10.8 (15)	25.2 (35)	11.5 (16)	13.7 (19)	38.9 (54)	44.6
	Updated	0.0 (0)	2.9 (4)	28.8 (40)	25.2 (35)	43.2 (60)	
Fruit juices (n= 193)	Original	0.0 (0)	25.4 (49)	21.8 (42)	22.3 (43)	30.6 (59)	77.7
	Updated	0.0 (0)	30.0 (58)	19.7 (38)	23.8 (46)	26.4 (51)	
Fruit juice concentrates and nectars (n= 916)	Original	0.0 (0)	6.2 (57)	39.4 (361)	23.7 (217)	30.7 (281)	82.1
	Updated	0.0 (0)	6.0 (55)	36.9 (338)	27.2 (249)	29.9 (274)	
Vegetable juices (n= 337)	Original	0.0 (0)	21.7 (73)	30.9 (104)	22.3 (75)	25.2 (85)	59.3
	Updated	0.0 (0)	19.3 (65)	47.2 (159)	19.6 (66)	13.9 (47)	
Overall (n= 3432)	Original	5.8 (200)	29.9 (1025)	41.6 (715)	17.7 (609)	25.7 (883)	
	Updated	0.0 (0)	15.8 (543)	33.0 (1132)	23.7 (812)	27.5 (945)	

Abbreviations: NS-NPM: Nutri-Score Nutrient Profiling Model.

Data are expressed in % (n).

The proportion of beverages is categorized into the five categories (A to E), based on the original or updated cut-offs for the respective beverage type.
